# Evaluating the Performance of ChatGPT4.0 Versus ChatGPT3.5 on the Hand Surgery Self-Assessment Exam: A Comparative Analysis of Performance on Image-Based Questions

**DOI:** 10.7759/cureus.77550

**Published:** 2025-01-16

**Authors:** Kiera L Vrindten, Megan Hsu, Yuri Han, Brian Rust, Heili Truumees, Brian M Katt

**Affiliations:** 1 Department of Orthopaedic Surgery, Rutgers Robert Wood Johnson Medical School, New Brunswick, USA

**Keywords:** ai, certification, chatgpt, education, self-assessment

## Abstract

Hypothesis

The emergence of ChatGPT as an artificial intelligence (AI) platform has become an increasingly useful tool in medical education, especially within resident education to supplement preparation for certification exams. As the AI model inevitably progresses, there is an increased need to establish ChatGPT’s accuracy in specialty knowledge. Our study assesses the performance of ChatGPT4.0 on self-assessment questions pertaining to hand surgery in comparison to the performance of its predecessor ChatGPT3.5. A distinct feature of ChatGPT4.0 is its ability to interpret visual input which ChatGPT3.5 cannot. We hypothesize that ChatGPT4.0 will perform better on image-based questions than ChatGPT3.5.

Methods

This study used 10 self-assessment exams from 2004 to 2013 from the American Society for Surgery of the Hand (ASSH). Performance on image-based questions was compared between ChatGPT4.0 and ChatGPT3.5. The primary outcome was the total score as a proportion of answers correct. Secondary outcomes were the proportion of questions for which ChatGPT4.0 provided elaborations, the length of those elaborations, and the number of questions for which ChatGPT4.0 provided answers with confidence. Descriptive analysis, Student's t-test, and one-way ANOVA tests were used for data analysis.

Results

Out of 455 image-based questions, there was no statistically significant difference in the total score between ChatGPT4.0 and ChatGPT3.5. ChatGPT4.0 answered 137 (30.1%) questions correctly while ChatGPT3.5 answered 131 (28.7%) correctly (p= 0.805). Although there was no significant difference in the length or frequency of elaborations in relation to the proportion of correct answers between the two versions, ChatGPT4.0 did provide significantly longer explanations overall compared to ChatGPT3.5 (p<0.05). Moreover, of the 455 total image-based questions, ChatGPT4.0 provided significantly less confident answers compared to ChatGPT3.5 (p<0.05). Of those responses in which ChatGPT4.0 expressed uncertainty, there was a significant difference based on image type, with the highest uncertainty stemming from question stems involving radiograph-based images (p<0.001).

Summary points

Overall, there was no significant difference in performance between ChatGPT4.0 and ChatGPT3.5 when answering image-based questions on the ASSH self-assessment examinations. Notably, however, ChatGPT4.0 expressed more uncertainty with answers. Further exploration of how AI-generated responses influence user behavior in clinical and educational settings will be crucial to optimizing the role of AI in healthcare.

## Introduction

Chat Generative Pre-trained Transformer (ChatGPT; OpenAI Incorporated, Mission District, San Francisco, United States) has rapidly emerged as a transformative technology that can stimulate human thought and reasoning [1]. The artificial intelligence (AI) platform uses a machine learning algorithm to generate human-like, coherent, and conversational responses by mapping relationships between context-based information [2]. ChatGPT has already made significant achievements in fields such as healthcare, where it assists in managing patient data, providing personalized information, and supporting medical education [1,3].

ChatGPT has been used in medical education to create automated scoring systems, personalized learning plans, and quizzes with detailed explanations [1], ultimately showing promise as a supplementary tool for student and resident education. Studies have already shown the remarkable performance of ChatGPT on medical school exams. One study assessed ChatGPT’s performance on Step 1, 2, and 3 exams, which was near the passing threshold without any specialized training [4] and was equivalent to that of a third-year medical student [5]. In addition to its notable performance on medical licensing examinations, ChatGPT’s performance has extended into different surgical specialties, including on neurosurgical written board exams [6] and the Orthopaedic In-Training Examination (OITE) [7], the latter of which is comparable to a first-year orthopedic surgery resident (PGY1) despite the overall unlikelihood of the platform passing the written examination. Other studies, such as that by Isleem et al., similarly demonstrated ChatGPT’s ability to perform at the level of an intern or junior orthopedic surgery resident on self-assessment exams from the American Academy of Orthopaedic Surgeons (AAOS) database and associated AAOS literature for the Orthopaedic Board Examination [8]. However, in many such studies, previous versions of ChatGPT, including ChatGPT3.5, were limited to interpreting solely text-based questions and lacked the ability to work with image-based content, which is a key aspect in many medical specialties such as orthopedic surgery.

The release of ChatGPT4.0 introduced enhanced capabilities, including the platform’s ability to interpret images, marking a significant advancement of the platform in medical education. This ability positions ChatGPT4.0 as a potentially valuable tool for specialty fields like orthopaedic hand surgery, where the accurate interpretation of visual content is essential. Despite these advancements, it remains crucial to assess whether ChatGPT4.0 can recognize its own limitations, particularly in high-stakes settings like patient care where reliance on AI-generated information can have serious consequences.

Confidence in AI-generated answers is important, as overly confident but incorrect responses can mislead users and spread misinformation. This was found to be the case with both ChatGPT3.5 and 4 when responding to radiology board-style multiple-choice questions [9]. An AI model that acknowledges its own limitations and provides uncertain answers when needed can encourage both trainees and educators to consult additional sources for more accurate information. This can enhance the reliability of AI as an educational tool and promote safer decision-making. Furthermore, evaluating whether ChatGPT4.0’s elaborations provide meaningful context when answering questions correctly can offer insights into its educational value.

This study aims to evaluate ChatGPT4.0’s performance on image-based questions from hand surgery self-assessment exams and compare it to ChatGPT3.5. We specifically investigate whether ChatGPT4.0 not only performs better than ChatGPT3.5 due to its visual interpretation capabilities but also if ChatGPT4.0 demonstrates improved recognition of its limitations. Understanding these factors will help determine the appropriate role of AI in medical education and its potential impact on learning outcomes and patient care.

## Materials and methods

This study utilized the American Society for Surgery of the Hand (ASSH) self-assessment exams from 2004 to 2013 to compare the performance of ChatGPT4.0 and ChatGPT3.5 on image-based questions. A total of 10 exams containing 1,605 questions were screened, of which 455 image-based questions were included after excluding text-based questions. These questions comprised various types of visual content, such as clinical images, radiographs, and anatomical diagrams relevant to hand surgery.

Each question, along with the corresponding image(s) (uploaded via screenshot), was manually copied and pasted into a ChatGPT4.0 web browser between December 28, 2023 to January 15, 2024 by a team of four medical students. To minimize potential biases, a site refresh was conducted before each question entry to prevent information carryover between ChatGPT responses. Questions were presented to ChatGPT without alteration, maintaining the original wording and images to consistently compare the two AI versions. These results using ChatGPT4.0 were then compared to previous data that was published by Han et al. using ChatGPT3.5 [10].

The primary outcome measured was the proportion of the correct answers provided by each ChatGPT version. Correct answers were defined as those matching the designated correct response from the ASSH exams. If ChatGPT gave the right answer or reported it could not answer a question but took a likely guess that ultimately was the correct answer, it was considered “correct.” If it gave the wrong answer or reported it could not answer an image-based question, it was considered “incorrect.”

Secondary outcomes included the frequency and length of elaborations. Elaboration was defined as the proportion of answers where ChatGPT provided additional explanation beyond simply stating the correct answer. The length of these elaborations was measured by the number of characters (including spaces), counted in Excel. Additionally, the level of confidence expressed by each version was categorized as either “confident” or “unconfident” based on whether the AI platform advised seeking further assistance from a medical professional.

Statistical analyses were performed using descriptive statistics, Student's t-tests, and one-way ANOVA tests to compare the correct answer proportions and confidence between ChatGPT4.0 and ChatGPT3.5. Differences in elaboration frequency were also analyzed to assess each version’s performance characteristics. All analyses were conducted using standard statistical software, and significance was defined at a p-value of less than 0.05.

## Results

A total of 455 image-based questions from 10 self-assessment exams provided by ASSH from 2004 to 2013 were analyzed. ChatGPT4.0 correctly answered 137 (30.1%) questions, while ChatGPT3.5 correctly answered 131 (28.7%). There was no statistically significant difference in performance between the two versions (Table 1, p= 0.805).

**Table 1:** ChatGPT4.0 v. ChatGPT3.5 performance on 455 total image-based questions (p=0.805, t stat=-0.250)

	ChatGPT3.5^10^	ChatGPT4.0
# correct	131 (28.8)	137 (30.1)
# incorrect	324 (71.2)	318 (69.9)
Score (%)	28.7	30.1

**Figure 1:**
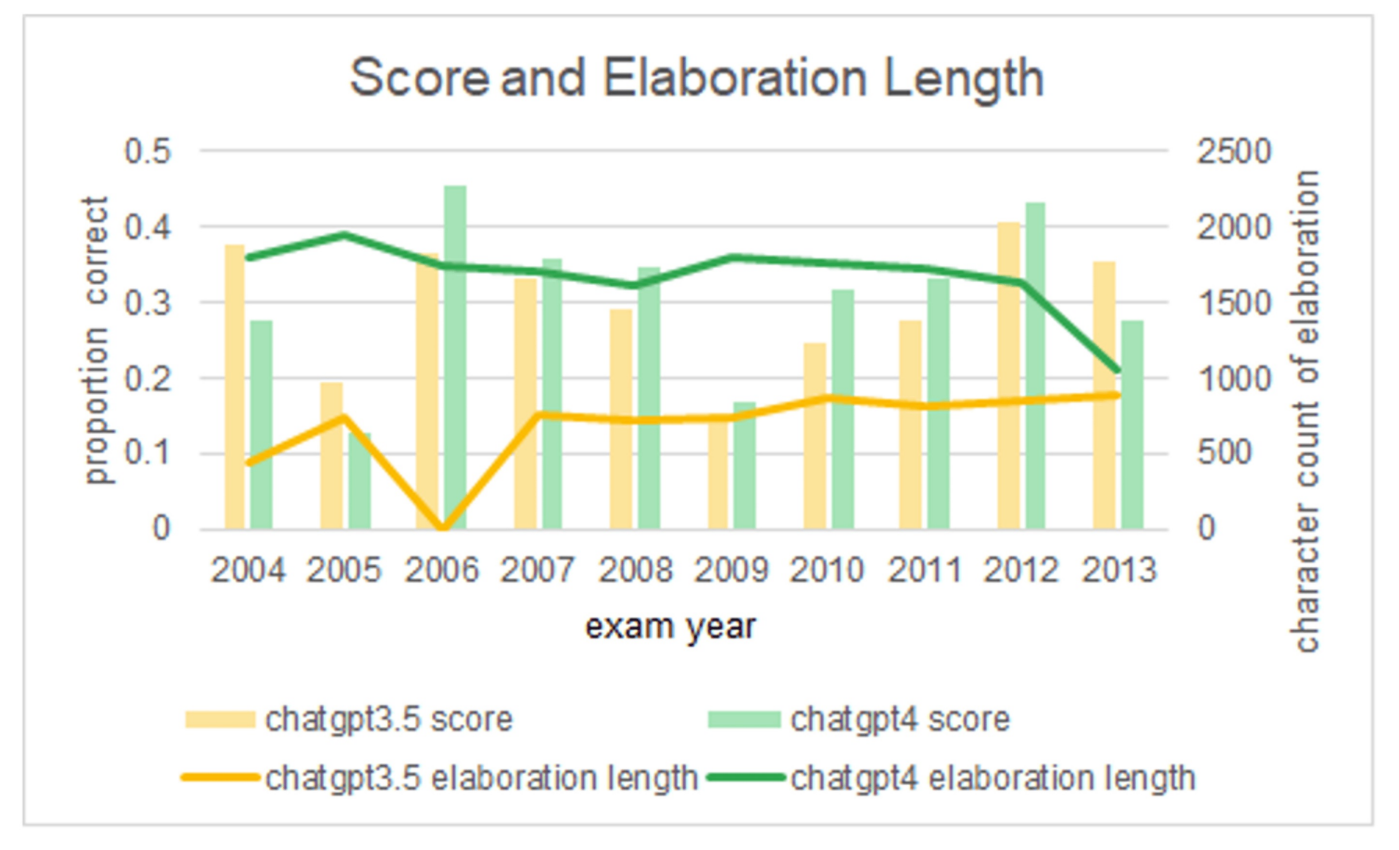
Performance and elaboration length between both versions of ChatGPT ChatGPT4.0 provided longer explanations than ChatGPT3.5 (P < 0.05, t stat=-7.73 for image-based questions only. ChatGPT3.5 data from the study by Han et al. [10].

When evaluating the number of questions for which answers with elaborations were provided, ChatGPT4.0 provided elaborations for 88.8% (404) of the questions, whereas ChatGPT3.5 did so for 69.9% (318) questions (Table 2, p=0.051), with ChatGPT4.0 providing significantly longer explanations compared to ChatGPT3.5 (Figure 1, p<0.05). However, there was no significant difference in the length or frequency of elaborations in relation to the proportion of correct answers between the two versions (Figure 1).

**Table 2:** ChatGPT4.0 versus ChatGPT3.5 performance on 455 total image-based questions (p = 0.051 and t stat=1.69 for elaborations; p<0.05 and t stat=9.13 for confident answers)

	ChatGPT3.5^10^	ChatGPT4.0
# elaborated answers (%)	318 (69.9)	404 (88.8)
# confident answers (%)	378 (88.1)	97 (21.3)

Notably, however, ChatGPT4.0 expressed unconfident answers in 78.7% of the questions, a substantially higher rate compared to ChatGPT3.5, which provided unconfident answers for only 11.9% of the questions (Table 2, p<0.05). Of those responses in which ChatGPT4.0 expressed uncertainty, there was a significant difference based on the image type, with the highest uncertainty stemming from question stems involving radiograph-based images (Table 3, p<0.001).

**Table 3:** ChatGPT4.0 uncertain responses by image type. The "mixed" category denotes question stems that incorporate more than one type of image. Percentage uncertain by each image type in parentheses. p< 0.001.

	Clinical Image	X-Ray	MRI	CT	Arteriogram	Anatomical Diagram	Mixed
2004	10 (83.3)	19 (86.4)	3 (100.0)	0 (-)	1 (100.0)	0 (-)	1 (50.0)
2005	13 (92.9)	8 (88.9)	3 (100.0)	0 (-)	0 (-)	0 (-)	3 (75.0)
2006	9 (100.0)	2 (100.0)	0 (-)	0 (-)	0 (-)	0 (-)	0 (-)
2007	17 (81.0)	14 (100)	1 (100.0)	0 (-)	0 (-)	0 (-)	2 (50.0)
2008	16 (72.7)	18 (81.8)	2 (100.0)	0 (-)	0 (-)	0 (0.0)	6 (75.0)
2009	19 (76.0)	15 (88.2)	4 (100)	1 (100.0)	0 (-)	0 (0.0)	6 (100.0)
2010	17 (63.0)	21 (84.0)	3 (100)	1 (100.0)	1 (100.0)	0 (-)	9 (69.2)
2011	26 (83.9)	19 (67.9)	0 (0.0)	0 (-)	0 (-)	0 (-)	12 (100)
2012	14 (82.4)	10 (83.3)	4 (100.0)	0 (-)	0(-)	0 (-)	4 (100.0)
2013	7 (82.4)	8 (53.3)	3 (100)	1 (100.0)	0 (0.0)	0 (0.0)	5 (41.7)
Total By Image Type (% of total # uncertain)	148 (41.3)	134 (37.4)	23 (6.4)	3 (0.8)	2 (0.6)	0 (0)	48 (13.4)

## Discussion

As ChatGPT becomes increasingly prevalent in the medical field as an instructional tool for medical education [11,12], it warrants a thorough investigation into the accuracy of the AI platform as new iterations like ChatGPT4.0 supersede predecessor versions like ChatGPT3.5. This study compared the performance of ChatGPT4.0 and ChatGPT3.5 on image-based questions from hand-surgery self-assessment exams, focusing on accuracy, elaborations, and expressed confidence levels. The results showed no significant difference in performance between the two versions, indicating that ChatGPT4.0’s enhanced visual interpretation capabilities did not translate into improved accuracy on these image-based questions.

Our results regarding ChatGPT4.0’s accuracy compared to ChatGPT3.5 contrast what has been shown in previous studies. One study using the ASSH Self-Assessment Examination (SAE) found that while examinees outperformed ChatGPT4.0 and 3.5, ChatGPT4.0 notably reduced the performance gap significantly and correctly answered more high-level questions than ChatGPT3.5 [13]. Similarly, another study demonstrated that ChatGPT4.0 exhibited an almost human-level performance in accuracy on the Medical Knowledge Self-Assessment Program (75% for GPT4, 53% for GPT3.5), although this study did not perform a sub-stratification analysis of image-based questions from text-only questions [14]. This performance trend with ChatGPT4.0 has also been found amongst other specialties as well, with another study reporting that ChatGPT4.0 outperformed ChatGPT3.5 on multiple choice urology board exam-style questions for specific subjects despite both platforms performing below passing rates [15]. However, our study results do align with those of Massey et al., which showed that, while ChatGPT4.0 correctly answered 35.7% of the image-based questions compared to ChatGPT3.5’s 22.4%, this difference was insignificant [16]. In short, the results of our study suggest that ChatGPT4.0 may not have superior performance over ChatGPT3.5, as previously shown for text-based questions, for image-based challenges.

This study also sought to evaluate the extent of elaboration of ChatGPT4.0 answers to image-based questions. Compared to previously published data [10], our results show that ChatGPT4.0 provided longer elaborations for image-based questions- almost doubled in size- than the length of elaboration provided by ChatGPT3.5. This finding could be explained by the nuanced advancements between ChatGPT3.5 compared with ChatGPT4.0, with ChatGPT4.0 likely acquiring more advanced model systems that are trained on larger, more elaborate datasets as well as possibly fine-tuning through the incorporation of human feedback. Thus, with more information to store in memory and use at its disposal, ChatGPT4.0 has the capacity to provide more detailed elaborations. It should be noted, however, that despite incorporating longer elaborations for image-based questions, this does not necessarily correlate with better performance or accuracy as demonstrated by the lack of significant difference in the length or frequency of elaborations between questions that ChatGPT4.0 answered correctly and incorrectly. This highlights the potential risk that ChatGPT4.0 could provide detailed, yet inaccurate explanations, which can ultimately mislead users. 

Another important finding from this study was the substantial increase in the rate of unconfident answers provided by ChatGPT4.0 compared to ChatGPT3.5, suggesting that ChatGPT4.0 may better recognize its own limitations. This finding starkly contrasts what was previously reported regarding the confidence of answers using the ChatGPT3.5 version, where ChatGPT3.5 provided confident answers 91.03% of the time, despite its low performance level (36.2%) [10]. This ability of ChatGPT4.0 to express uncertainty can guide users to seek additional verification from other reliable sources rather than depending solely on AI-generated responses, ultimately enhancing the platform’s use as a supportive tool in medical education and patient care. Moreover, while this data confirms that the AI platform is doing a better job of encouraging its users to seek advisement from medical professionals before solely relying on the information it provides, this reaffirms the idea that all users of ChatGPT, medical professionals and trainees, as well as patients and their families, should continue to use these educational tools with caution.

This study has several limitations that should be noted. While each image-based question within the ASSH self-assessment examinations from 2004 to 2013 was input following the same protocol, the data was collected over a span of multiple days and amongst multiple co-investigators. In doing so, the performance of ChatGPT4.0 is subject to limitations involving internet traffic and connectivity, as previously described [10]. Furthermore, another limitation of ChatGPT that must be considered is that the platform is not only biased toward the validity of the data that it is trained on but also in that the extent of the training data included is only up to a certain point in time for each iteration of ChatGPT with no real-time updates. Additionally, this training does not necessarily translate into ChatGPT’s capability of higher-order critical thinking. This was observed through recent studies which demonstrated the failure of ChatGPT to reach the passing threshold of the American Heart Association (AHA) Advanced Cardiovascular Life Support (ACLS) and Orthopaedic Fellow of the Royal College of Surgeons Part A exam due to its robustness and limited critical thinking [17-19]. Another study reported similar findings, noting that ChatGPT reported better on questions requiring lower-order thinking when tasked with answering radiology board-style examinations [20]. Thus, even as ChatGPT is trained on new data with each iteration, it remains limited in its critical thinking skills that many of these medical exams require of examinees. Lastly, since ChatGPT4.0 provides unique answers to the same questions, future research should investigate and consider the intra-variability that exists between ChatGPT4.0 responses when answering the same image-based question at separate time points.

## Conclusions

In sum, there was no significant difference in performance accuracy for answering image-based questions on the ASSH self-assessment examinations when comparing ChatGPT4.0 to ChatGPT3.5. Notably, however, ChatGPT4.0 expressed more unconfident answers, indicating that it may better recognize its limitations compared to earlier AI versions. Despite its enhanced image-interpretation capabilities, ChatGPT4.0 has not yet achieved sufficient proficiency in hand sub-specialty image analysis to support clinical diagnosis independently.
